# Epigenetic Epidemiology of Common Complex Disease: Prospects for Prediction, Prevention, and Treatment

**DOI:** 10.1371/journal.pmed.1000356

**Published:** 2010-10-26

**Authors:** Caroline L. Relton, George Davey Smith

**Affiliations:** 1 Human Nutrition Research Centre, Institute of Human Genetics, Newcastle University, Newcastle upon Tyne, United Kingdom; 2 MRC Centre for Causal Analyses in Translational Epidemiology, School of Social and Community Medicine, University of Bristol, Bristol, United Kingdom

## Abstract

As part of the PLoS Epigenetics Collection, Caroline Relton and George Davey Smith discuss the potential of epigenetics for the treatment and prevention of common complex diseases, including cancer.

**Summary Points**

- The epigenome records a variety of dietary, lifestyle, behavioral, and social cues, providing an interface between the environment and the genome. Epigenetic variation, whether genetically or environmentally determined, contributes to inter-individual variation in gene expression and thus to variation in common complex disease risk.
- Interventions based upon epigenetic agents, including DNA methyltransferase inhibitors and histone deacetylase inhibitors, have been in clinical use for many years, but their role outside treatment of specific cancers is not established.
- Epigenetic therapies will only be fruitful if epigenetic mechanisms are causally related to the disease being treated. Evidence linking epigenetic variation to specific disease phenotypes to date is lacking.
- Epidemiological approaches can be applied to help separate causal from non-causal associations.
- We propose the development of a Mendelian randomization approach (“genetical epigenomics”), which could help overcome the problems of confounding and reverse causation (when an association between epigenetic patterns and disease phenotype is observed but it is unknown whether the disease is causing changes to the epigenome or epigenetic changes are causal in disease pathogenesis).

## Introduction

There is considerable anticipation of future improvements in disease prevention and treatment following recent advances in genomics [1]. One aspect of genomics that is receiving considerable interest is epigenetics—the regulatory processes that control the transcription of information encoded in the DNA sequence into RNA before their translation into proteins. Programmed developmental changes and the ability of the genome to register, signal, and perpetuate environmental cues are subsumed under the epigenetic banner [2].

**Figure 1:**
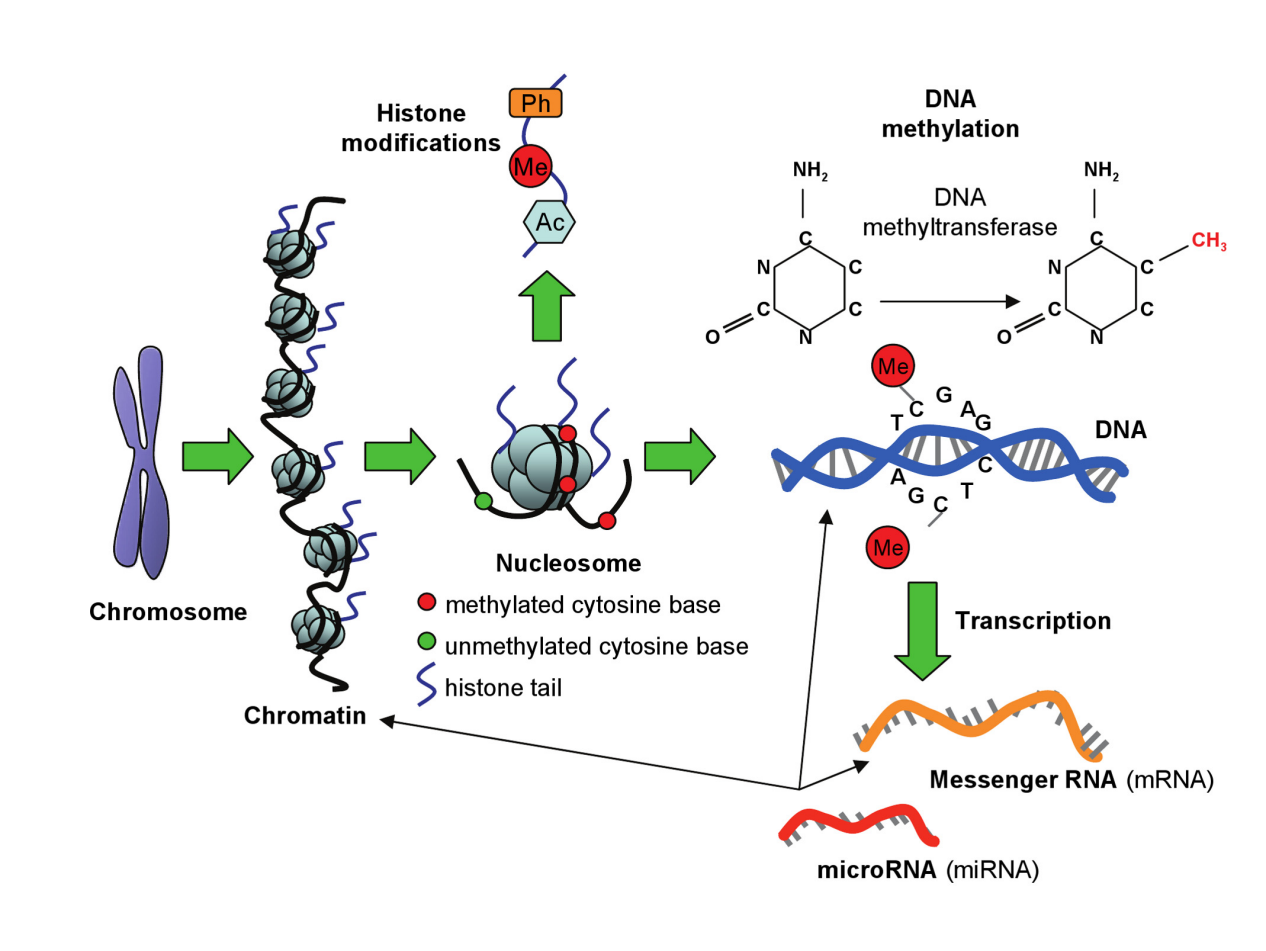
Epigenetic modifications. Chromosomes are composed of chromatin, consisting of DNA wrapped around eight histone protein units. Each DNA-bound histone octamer is a nucleosome. Histone tails protruding from histone proteins are decorated with modifications, including phosphorylation (Ph), methylation (Me), and acetylation (Ac). DNA molecules are methylated by the addition of a methyl group to carbon position 5 on cytosine bases when positioned adjacent to a guanine base (CpG sites), a reaction catalyzed by DNA methyltransferase enzymes. DNA methylation maintains repressed gene activity. Transcription involves the conversion of DNA to messenger RNA (mRNA), which is usually repressed by DNA methylation and histone deacetylation. mRNA is translated into a protein product, but this process can be repressed by binding of microRNA (miRNA) to mRNA. Each miRNA binds to the mRNA of up to 200 gene targets. miRNAs can also be involved in establishing DNA methylation and may influence chromatin structure by regulating histone modifiers.

Genes are packaged into chromatin and dynamic chromatin remodeling processes are required for the initial step in gene expression (transcription), achieved by altering the accessibility of gene promoters and regulatory regions [3]. Epigenetic factors are responsible for this regulatory process, the major components of which are DNA methylation, histone modifications, and the action of small non-coding RNAs (Figure 1). Unlike DNA sequence, which is largely fixed throughout the lifecourse, epigenetic patterns not only vary from tissue to tissue but alter with advancing age and are sensitive to environmental exposures [4]–[7]. It is this propensity for change that makes epigenetic processes the focus of such interest, as they lie at the interface of the environment and co-ordinated transcriptional control.

In rare developmental disorders, the role of aberrant epigenetic processes is well established [8]. Our focus here, however, is on the potential role of epigenetic processes in the context of common complex disease. Tumor-specific changes in epigenetic patterns are a hallmark of numerous cancers, with analysis of the epigenetic machinery beginning to feature prominently in emerging cancer diagnostics and therapies [9]–[11].

There is an increasing body of evidence to demonstrate that epigenetic patterns are altered by environmental factors known to be associated with disease risk (e.g., diet, smoking, alcohol intake, environmental toxicants, stress) [7],[8]; however, an important question remains to be resolved in defining which epigenetic changes are a secondary outcome of either exposure or disease, and which lie on the causal pathway linking the two. Without proven causality, interventions to prevent or treat common complex diseases based upon epigenetic mechanisms will not be fruitful. Conversely, regardless of causality, defining a robust prospective relationship between epigenetic patterns and phenotypic traits may have application in diagnostics or in identifying high-risk individuals for non-epigenetic-based interventions.

## Measurement of Epigenetic Patterns

Epigenetic patterns, including histone modifications, microRNA (miRNA), and DNA methylation, can be assessed in a range of tissue types. As DNA methylation assays on stored DNA samples are straightforward, this has been extensively studied [12]. Histone modification analysis requires that DNA is maintained as intact chromatin, whereas analysis of miRNA requires a source of RNA. Planned prospective collection for such analyses is necessary, and both are costly to undertake on sizable sample sets. The N-terminal tails of the four core histones (H2A, H2B, H3, and H4) commonly exhibit post-translational modifications, including acetylation, methylation, or phosphorylation [13]. These histone modifications can be analysed following precipitation of chromatin, and subsequent use of an antibody to a specific modification e.g., methylation of histone 3, lysine 9 (H3-K9). miRNA expression levels can be measured using the same principles and methods as regular trranscriptomic analysis (miRNA array or qPCR). DNA methylation can be assayed through genome-wide approaches where the investigator is interested in global changes or in identifying regions of interest [14], or targeted approaches that focus on DNA methylation at a particular locus or loci associated with genes in a specific pathway [15]. These technologies are reviewed in detail elsewhere [16].

The tissue specificity of epigenetic patterns is a well-established phenomenon, with variation between tissues within individuals being greater than variation between individuals [5]. Furthermore, epigenetic dysregulation with advancing age has been shown to be highly tissue dependent [17]. Extrapolating epigenetic information gleaned from DNA from accessible sources such as peripheral white blood or buccal cells to other tissue types is therefore problematic. The correlation between methylation patterns in different tissues is complex and locus dependent, but data that are beginning to emerge suggest that epigenetic signatures on easily accessible material such as circulating cells have potential utility as biomarkers of exposure or disease risk [18].

Epigenetic patterns are heritable across cell divisions (mitosis) [19], but undergo comprehensive but incompletely understood reprogramming during meiosis [20]. Evidence that environmental exposures can act across generations to influence epigenetic patterns in offspring exist [21], with maternal exposure to famine during the perinatal period influencing offspring DNA methylation in adulthood [22],[23]. The quantitative importance of such intergenerational epigenetic transmission remains uncertain, and may have been over-emphasized in comparison with the theoretically less challenging but probably more tractable and important intra-generational epigenetic influences [24].

## Environmental Influences on Epigenetic Patterns

Several other factors beyond tissue type and age [4],[5],[17],[25],[26] are believed to influence epigenetic patterns. Nutritional factors modulate epigenetic marks in both animal models and humans (reviewed by [27]), with dietary sources of methyl groups, including folate, choline, betaine, methionine, and serine, which are required for DNA methylation [28],[29], having been most studied. In animal and human studies these modulate epigenetic patterns in disease and non-disease settings. Other dietary components with evidence for an effect on epigenetic patterns relevant to the pathogenesis of common complex diseases include the influence of a high-fat diet on DNA methylation [30] and various dietary modifiers of histone deacetylase (HDAC) activity such as isothiocyanates, butyrate, and diallyl disulfide [31],[32]. miRNA levels have also been observed to be altered following dietary modulation, with miRNA expression in human muscle being increased following a dietary challenge of essential amino acids [33].

The most widely studied lifestyle influence on epigenetic patterns is smoking. It has been associated with global hypomethylation in DNA [34] as well as gene-specific hypermethylation [35] in tumor tissues in head and neck squamous cell carcinoma (HNSCC). Animal models suggest that epigenetic changes arise in lung tissue following short-term exposure to tobacco smoke condensate [36] and precede histopathological changes. Exposure to tobacco smoke is also believed to alter expression of DNA methyltransferase (DNMT) enzymes [37],[38] and modulate histone modifications, including acetylation and methylation [39]. In addition, miRNAs have been proposed as modulators of smoking-induced changes in gene expression in human airway epithelium [40], and studies in rodent models have demonstrated that chemopreventive agents can protect the lung tissue from smoke exposure-induced changes in miRNA expression [41]. Maternal cigarette smoking during pregnancy influences DNA methylation patterns in offspring [42],[43], pointing to a vulnerability of the epigenome to environmental exposures during the intrauterine period.

Animal studies have shown that chronic alcohol consumption is associated with reduced genomic DNA methylation in the colon [44], although evidence from human studies is equivocal. Alcohol-induced shifts in DNA methylation patterns could arise through perturbation of one-carbon metabolism and interference with methyl group donation (reviewed by [45]). The molecular actions of ethanol are also thought to involve site-specific changes to histone modifications, exemplified by a recent study of alcohol exposure during adolescence [46]. Epigenetic processes could also influence patterns of alcohol drinking, with emerging evidence suggesting that alcohol-sensitive miRNAs control the development of tolerance and subsequent alcohol addiction [47]. The alcohol-related miRNA responses may in turn reflect alcohol-induced changes in DNA methylation [48].

Air pollutants such as air particulate matter and airborne benzene exposure levels have been associated with changes in DNA methylation in genes involved in inflammation and carcinogenesis [49],[50]. Endocrine disruptors (vinclozilin, bisphenol A), and various heavy metals (arsenic, mercury, cadmium) are among other compounds present in the environment that have been implicated in epigenetic changes, including altered histone methylation [21]. Most epigenetic studies of environmental toxins have focused on the potential of DNA methylation patterns as biological markers of exposure rather than establishing epigenetic mechanisms as being causally related to a specific disease. Studies have, however, suggested a role for miRNAs in mediating the effects of exposure to black carbon on disease [51].

Several infectious agents, including *Helicobacter pylori* [52] and Epstein-Barr virus [53], have been shown to induce epigenetic changes, either directly or secondary to inflammation. Epigenetic modulation is recognized as an aetiological component in chronic inflammatory diseases such as rheumatoid arthritis and multiple sclerosis [54]. Inflammation also plays an important role in a wide range of diseases such as cancers, obesity, and atopic disorders, and epigenetic changes may be causal in disease pathogenesis [54]. There is increasing evidence that epigenetic mechanisms contribute to the transcriptional regulation of inflammatory responses [55].

Perhaps the most widely celebrated example of the influence of environmental conditions (other than diet) on the epigenome relates to maternal postnatal nurturing and epigenetically mediated alterations to the hypothalamic-pituitary-adrenal response to stress [56]. Variations in maternal signals alter gene expression and complex behavioral phenotypes in rodent offspring through a well-defined mechanism involving the epigenetic regulation of the glucocorticoid receptor gene within the target tissue. A further example of modulation of epigenetic patterns in a target tissue is that of increased histone acetylation in human muscle biopsy tissue following exercise [57], providing evidence that chromatin remodeling might be important in mediating longer-term responses to exercise. miRNA involvement in exercise-induced changes to gene expression has also been reported [58].

## Genetic Influences on Epigenetic Patterns

Twin- and family-based studies have demonstrated that variation in epigenetic patterns, including both chromatin states [59] and DNA methylation [25],[60],[61], is heritable. Much inter-individual variation in epigenetic patterns can be explained by common genetic variation [62], with a recent study estimating that 6.5% of the variance in methylation at the *IGF2* (insulin-like growth factor 2) locus could be explained by five single nucleotide polymorphisms (SNPs) [63]. A genome-wide association study considering DNA methylation in human brain tissue as a quantitative trait identified both *cis* and *trans* genetic effects upon DNA methylation (cytosine guanine dinucleotide [CpG]) sites, the predominant influences being in *cis*, defined as SNPs influencing methylation at CpG sites within 1 Mb of themselves [64]. Similar *cis* effects have been reported in whole blood DNA [25]. Greater knowledge of the genetic determinants of DNA methylation, histone modifications, and miRNA activity will transform our understanding of the mechanisms involved in the establishment and maintenance of epigenetic patterns, with such genetic influences undoubtedly contributing to observed inter-individual differences in gene expression [65].

**Figure 2:**
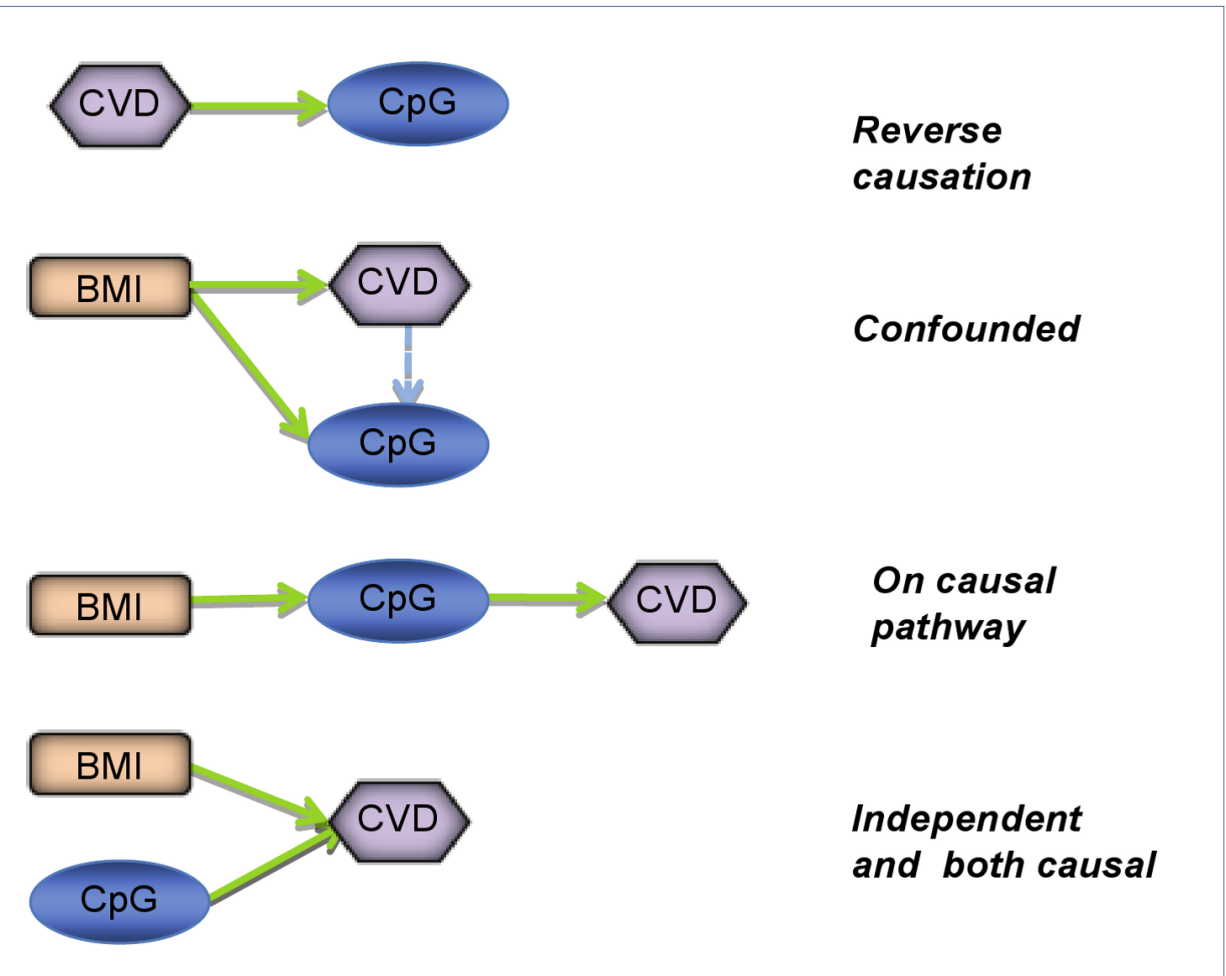
Defining the causal relationship between epigenetic patterns and phenotype. Analysis of the respective relationships between DNA methylation (CpG), body mass index (BMI), and cardiovascular disease (CVD) can help to inform the direction of causality. An observed association between BMI and CpG and CpG and CVD will not decipher which of the depicted scenarios apply.

Despite the relatively large body of evidence that disease-related environmental exposures are associated with epigenetic alterations, there remains little compelling data to support the link between epigenetic variation and common complex disease phenotypes (other than cancer). Investigation of parent-of-origin effects on risk of common complex disease have suggested a role of perturbed DNA methylation [66]. Adequately powered studies relating epigenetic profiles to both exposure and disease are in their infancy, but it is highly likely that a myriad of such associations will be identified, and the major issue will be identifying meaningful and useful associations within this tsunami of data. Epigenetic measures are phenotypic, not genotypic, and as with phenotypic measures in general, non-causal associations will be the rule rather than the exception [67]. As with conventional epidemiological investigations, separating causal from non-causal associations will become an important task (Figure 2).

## “Genetical Epigenomics”: Identifying Causal Relationships between Exposure, Epigenetic Patterns, and Disease

Using germ-line genetic variation as a proxy for environmental exposures provides a route to strengthening causal inference within observational data [68]–[70]. The rationale is that genetic variants are not, in general, related to the socio-economic, behavioral, and physiological factors that confound associations in conventional observational epidemiology [67], nor are they altered by disease processes and thus subject to reverse causation. The Mendelian randomization approach can be extended to the interrogation of epigenetic variation as potential mediators of the influence of a modifiable exposure on disease outcomes, and thus appropriate targets for disease prevention.

**Figure 3:**
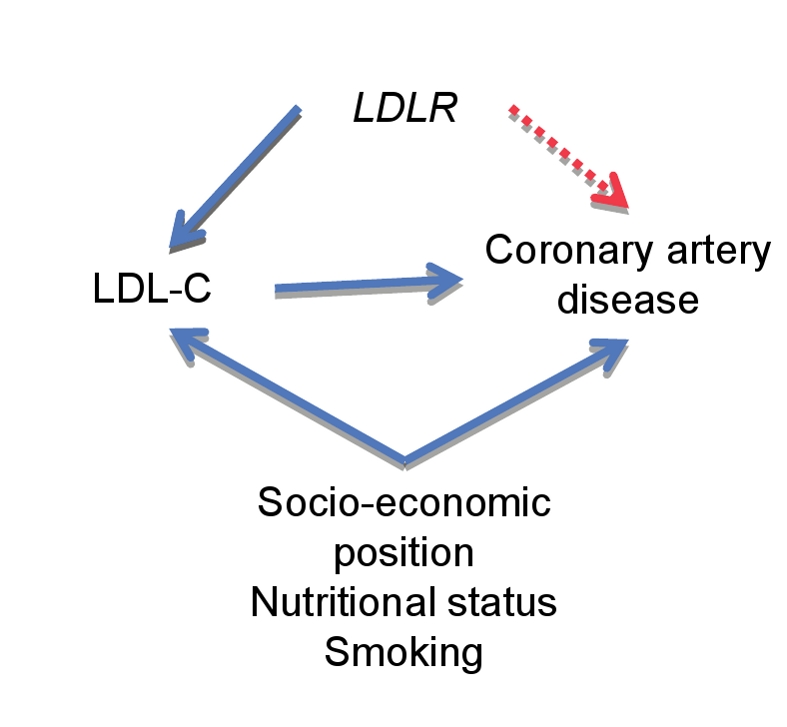
Applying Mendelian randomization to define the causal relationship between phenotype and disease. An example based upon the report of Lintel-Nietschke et al. (2008) [74] reporting the association between a gene variant in the *LDLR* gene with decreased low density lipoprotein-cholesterol (LDL-C) levels and with a reduced risk of coronary artery disease (CAD). The variant can be used in a Mendelian randomization approach to test the causal relationship between LDL-C and CAD. If LDL-C has a causal role in CAD, an association between the *LDLR* gene variant and disease risk would be seen (red dashed arrow). If LDL-C levels are correlated with CAD risk but not causal, then the gene variant will not show an association with CAD risk. This will establish whether reverse causation is at play and remove the potential confounding influence of factors such as smoking and nutritional status.

Mendelian randomization methods can be applied to many categories of environmentally modifiable exposures to help define whether their relationship with phenotype is causal. For example, with respect to behavioral factors, it has been used in a proof-of-principle manner to demonstrate associations of alcohol intake with esophageal [71] and head and neck cancers [72], as well as to considerably strengthen evidence on the associations of alcohol intake with blood pressure [73]. The method has particular promise when applied to circulating intermediate phenotypes, the manipulation of which can potentially prevent disease. Again, as proof-of-principle, an increasing number of genetic variants that are associated with low density lipoprotein-cholesterol (LDL-C) level are also associated with coronary artery disease (CAD) risk [67],[74]–[76] (Figure 3).

In a similar fashion, genetic variants related to body mass index and obesity have been shown to influence a wide variety of metabolic, cardiovascular, and bone-related traits, strengthening evidence on the causal influence of adiposity in these cases [77]–[80]. Conversely, genetic variants associated with C-reactive protein (CRP) level have not been found to predict insulin resistance [80] or coronary heart disease [81], casting doubt on the causal role of CRP with respect to these conditions.

In the field of gene expression studies, identifying causal processes within a multitude of associations is at least as problematic as in observational epidemiological studies. For example, the majority of gene expression signatures in adipose tissue, and in high proportions (up to 10%) in blood, have been found to be related to obesity [82]. Methods equivalent to the Mendelian randomization approach we propose here (sometimes called “genetical genomics” [83] in the context of gene expression studies) have been applied to separate causal transcription effects from those generated by reverse causation [82]. This is facilitated by strong *cis* effects on gene expression, which allows isolation of specific loci influencing transcript level. The identification of strong *cis* effects in a genome-wide association study analysis of methylation patterns [64] provides encouragement that these methods can be extended to investigate the causal influences of epigenetic signatures in what could be called “genetical epigenomics”.

**Figure 4:**
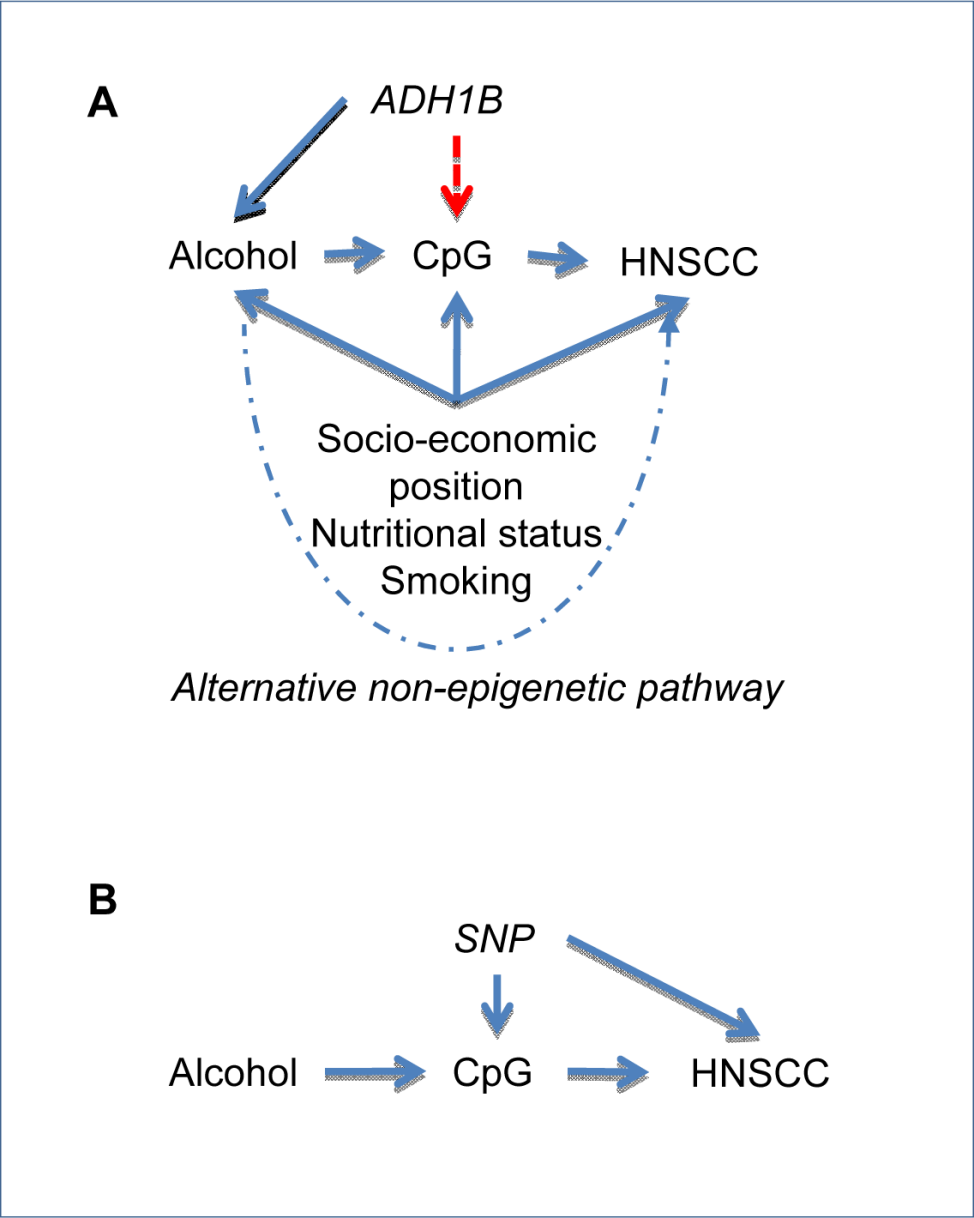
Incorporating epigenetic information in a Mendelian randomization framework. (A) Alcohol exposure is associated with risk of head and neck squamous cell carcinoma (HNSCC) and this may be mediated by altered DNA methylation (CpG). The relationship between alcohol exposure and HNSCC is potentially confounded by factors such as socio-economic position, which correlate with both exposure and disease. A common variant in ADH1B can be used as an unconfounded, genetic proxy for alcohol exposure, and if this SNP is associated with CpG (either locally or more widely across the genome), it would lend support to the hypothesis that alcohol intake causally influences DNA methylation. However, showing associations of these epigenetic measures with HNSCC does not demonstrate causality of either alcohol or CpG on HNSCC, as either or both associations (alcohol→HNSCC and CpG→HNSCC) could be confounded or alcohol could influence HNSCC through another pathway (dashed line). (B) To investigate this, another Mendelian randomization experiment could be undertaken using an SNP known to have a *cis* influence on loci-specific DNA methylation. If an association were observed between this SNP and both CpG and HNSCC, this would support a role for DNA methylation in the causation of HNSCC.

As a hypothetical example of how this approach could be applied, we will consider alcohol intake and HNSCC. It is likely that alcohol intake would be associated with a wide range of epigenetic changes, although at least some (and probably many) of these associations could reflect confounding by the many other factors related to alcohol consumption. Similarly, HNSCC could be related to a multitude of epigenetic changes, which could arise through reverse causation (the disease influencing the epigenetic patterns) or confounding (factors associated with HNSCC risk influencing the epigenetic patterns). If the epigenetic processes are to be targeted as a component of disease prevention they must be causally associated with HNSCC, and for them to mediate the effect of alcohol intake on HNSCC risk they need to be responsive to changes in alcohol intake. Observational data demonstrating an association of alcohol intake with a particular epigenetic profile exists, but the association of this profile with HNSCC risk does not, of course, establish causality. As depicted in Figure 4, Mendelian randomization approaches could be applied to this scenario.

## Epigenomic Modifiers and the Prospects for Future Treatments

It can be argued that mitotically stable changes in gene expression are very likely to underlie the development of virtually all disease (in the same way as they are an essential component in the process of the development of an organism [84]), and as definitions of epigenetics incorporate such changes, they automatically fall within the field's remit. Once epigenetic mechanisms, even if only contributory, are unequivocally implicated in disease pathogenesis, the prospect of epigenomic-based therapies becomes a realistic possibility. A wide range of pharmacological agents that target the epigenome, including DNMT inhibitors and HDAC inhibitors, are used in clinical practice, largely as anti-cancer treatments [11]. However, these agents require further development to enhance the specificity of their pleiotropic effects, and evaluation of their efficacy in a non-cancer setting is essential. Combination therapies involving DNMT inhibitors or HDACs being employed with other agents are an active avenue of inquiry. miRNAs are also emerging as a promising technology in drug development following an increasing understanding of their biogenesis and function. The links between miRNA expression and common complex disease are growing, providing a greater impetus to pursue this useful tool for the targeted modulation of gene regulation. As with other epigenetic signatures, their utility might also lie in disease diagnosis and prognosis [85].

## Conclusion

Through examining the role of environmental factors in causing variation in epigenetic patterns (exposure/epigenotype) and ultimately exploring the causal impact of epigenotype on disease outcomes (epigenotype/disease) using genetical epigenomics and other methods, progress towards epigenetic interventions can be made. As genome-wide association studies and other approaches identify robust associations between genetic variants and epigenetic patterns, possibilities for elucidating causal pathways and predicting the effect of manipulation—through environmental (including lifestyle) modification or pharmacotherapeutic means—is considerable. In this way, epigenetic markers may become targets for modification as well as biomarkers for exposure and disease risk. The International Human Epigenome Consortium is poised to invest millions of dollars to map 1,000 reference epigenomes in a range of normal tissues and define the level of variation that exists between individuals [86]. The field of epigenetics in relation to common complex disease will undoubtedly continue to be the focus of much attention, and its progress, now that it has passed the starting line, will be followed with considerable interest.

**Five Key Papers in the Field**

- Weaver IC, Cervoni N, Champagne FA, D'Alessio AC, Sharma S, et al. (2004) Epigenetic programming by maternal behaviour. Nat Neurosci 7: 847–854. This landmark paper demonstrated that the epigenomic state of a gene can be altered through behavioural programming and that this environmentally induced modification is potentially reversible.
- Fraga MF, Ballestar E, Paz MF, Ropero S, Setien F, et al. (2005) Epigenetic differences arise during the lifetime of monozygotic twins. Proc Natl Acad Sci U S A 102: 10604–10609. This article describes how epigenetic patterns in monozygotic twins become more discordant with advancing age. This epigenetic drift is postulated to be invoked through differences in environmental exposures.
- Bjornsson HT, Sigurdsson MI, Fallin MD, Irizarry RA, Aspelund T, et al. (2008) Intra-individual change over time in DNA methylation with familial clustering. JAMA 299: 2877–2883. This study showed greater than 10% methylation change over time, that individuals within families showed both gain and loss of methylation, and that this change in methylation showed familial clustering indicative of a genetic basis.
- Lister R, Pelizzola M, Dowen RH, Hawkins RD, Hon G, et al. (2009) Human DNA methylomes at base resolution show widespread epigenomic differences. Nature 462: 315–322. This paper reports the first genome-wide, single base-pair resolution map of methylated cytosines in the mammalian genome from embryonic stem cell and fetal fibroblasts, showing widespread differences between the tissue types.
- Zhang D, Cheng L, Badner JA, Chen C, Chen Q, Luo W, et al. (2010) Genetic control of individual differences in gene-specific methylation in human brain. Am J Hum Genet 86: 411–419. This study demonstrated that DNA methylation is a heritable trait, determined in part by common genetic variation. The vast majority of genetically determined variation was observed to be in *cis* (correlation within 1Mb of a CpG site) with only a handful of SNPs determining *trans* methylation (distant regulation effects).

## Abbreviations

CAD: coronary artery disease
CpG: cytosine guanine dinucleotide
DNMT: DNA methyltransferase
HDAC: histone deacetylase
HNSCC: head and neck squamous cell carcinoma
LDL-C: low density lipoprotein-cholesterol
microRNA: miRNA
SNP: single nucleotide polymorphism

## References

[1] Feero WG, Guttmacher AE, Collins FS (2010). Genomic medicine–An updated primer.. New Engl J Med.

[2] Bird A (2007). Perceptions of epigenetics.. Nature.

[3] Vaissiere T, Sawan C, Herceg Z (2008). Epigenetic interplay between histone modifications and DNA methylation in gene silencing.. Mutat Res.

[4] Lister R, Pelizzola M, Dowen RH, Hawkins RD, Hon G (2009). Human DNA methylaomes at base pair resolution show widespread epigenomic differences.. Nature.

[5] Byun HM, Siegmund KD, Pan F, Weisenberger DJ, Kanel G (2009). Epigenetic profiling of somatic tissues from human autopsy specimens identifies tissue- and individual-specific DNA methylation patterns.. Hum Mol Genet.

[6] Aguilera O, Fernandez AF, Munoz A, Fraga MF (2010). Epigenetics and environment: a complex relationship.. J Appl Physiol.

[7] Meaney MJ (2010). Epigenetics and the biological definition of gene x environment interactions.. Child Dev.

[8] Nicholls RD (2000). The impact of genomic imprinting for neurobehavioural and developmental disorders.. J Clin Invest.

[9] Sharma S, Kelly TK, Jones PA (2010). Epigenetics in cancer.. Carcinogenesis.

[10] Laird PW (2003). The power and the promise of DNA methylation markers.. Nat Rev Cancer.

[11] Piekarz RL, Bates SE (2009). Epigenetic modifiers: Basic understanding and clinical development.. Clin Cancer Res.

[12] Beck S, Rakyan VK (2008). The methylome: approaches for global DNA methylation profiling.. Trends Genet.

[13] Jenuwein T, Allis CD (2001). Translating the histone code.. Science.

[14] Feinberg AP (2009). Genome-scale approaches to the epigenetics of common human disease.. Virchows Arch.

[15] Campion J, Milagro FI, Martinez JA (2009). Individuality and epigenetics in obesity.. Obes Rev.

[16] Tollefsbol TO, Tollefsbol TO (2004). Methods of epigenetic analysis.. Epigenetics protocols.

[17] Thompson RF, Atzmon G, Gheorghe C, Liang HQ, Lowes C (2010). Tissue specific dysregulation of DNA methylation in aging.. Aging Cell.

[18] Talens RP, Boomsa DI, Tobi EW, Kremer D, Jukema JW (2010). Variation, patterns and temporal stability of DNA methylation: considerations for epigenetic epidemiology.. FASEB J.

[19] Kim JK, Samaranayake M, Pradhan S (2009). Epigenetic mechanisms in mammals.. Cell Mol Life Sci.

[20] Reik W, Dean W, Walter J (2001). Epigenetic reprogramming in mammalian development.. Science.

[21] Bollati V, Baccarelli A (2010). Environmental epigenetics.. Heredity.

[22] Heijmans BT, Tobi EW, Stein AD, Putter H, Blauw GJ (2008). Persistent epigenetic differences associated with prenatal exposure to famine in humans.. Proc Natl Acad Sci U S A.

[23] Tobi EW, Lumey LH, Talens RP, Kremer D, Putter H (2009). DNA methylation differences after exposure to prenatal famine are common and timing- and sex-specific.. Hum Mol Genet.

[24] Haig D (2007). Weismann rules! OK? Epigenetics and the Lamarckian temptation.. Biol Philos.

[25] Boks MP, Derks EM, Weisenberger BJ, Strengman E, Janson E (2009). The relationship of DNA methylation with age, gender and genotype in twins and healthy controls.. PLoS ONE.

[26] Calvanese V, Lara E, Kahn A, Fraga MF (2009). The role of epigenetics in ageing and age-related diseases.. Ageing Res Rev.

[27] Ferguson LR (2009). Epigenetic variation and customising nutritional intervention.. Curr Pharmacogenomics Person Med.

[28] Kim KC, Friso S, Choi SW (2009). DNA methylation, an epigenetic mechanism connecting folate to healthy embryonic development and aging.. J Nutr Biochem.

[29] Waterland RA (2006). Assessing the effects of high methionine intake on DNA methylation.. J Nutr.

[30] Widiker S, Karst S, Wagener A, Brockman GA (2010). High fat diet leads to a decreased methylation of the Mc4r gene in the obese BFMI and the lean B6 mouse lines.. J Appl Genet.

[31] Delage B, Dashwood RH (2008). Dietary manipulation of histone structure and function.. Annu Rev Nutr.

[32] Myzak MC, Dashwood RH (2006). Histone deacetylases as targets for dietary cancer preventive agents: lessons learned with butyrate, diallyl disulfide and sulforaphane.. Curr Drug Targets.

[33] Drummond MJ, Glynn EL, Fry CS, Dhanani S, Volpi E (2009). Essential amino acids increase miRNA-499, -208b and -23 in human skeletal muscle.. J Nutr.

[34] Hsiung DT, Marsit CJ, Houseman EA, Eddy K, Furniss CS (2007). Global DNA methylation level in whole blood as a biomarker in head and neck squamous cell carcinoma.. Cancer Epidemiol Biomarkers Prev.

[35] Kaur J, Demokan S, Tripathi SC, Macha MA, Begum S (2010). Promoter hypermethylation in indian primary oral squamous cell carcinoma.. Int J Cancer.

[36] Philips JM, Goodman JI (2009). Inhalation of cigarette smoke induces regions of altered DNA methylation (RAMs) in SENCAR mouse lung.. Toxicology.

[37] Launay JM, Del Pino M, Chironi G, Callebert J, Peoc'h K (2009). Smoking induces long-lasting effects through a monoamine-oxidase epigenetic regulation.. PLoS ONE.

[38] Liu H, Zhou Y, Boggs SE, Belinsky SA, Liu J (2007). Cigarette smoke induces demethylation of prometastatic oncogene synuclein-gamme in lung cancer cells by downregulation of DNMT3B.. Oncogene.

[39] Hussain M, Rao M, Humphries AE, Hong JA, Liu F (2009). Tobacco smoke induces polycomb-mediated repression of Dickkopf-1 in lung cancer cells.. Cancer Res.

[40] Schembri F, Sridhar S, Perdomo C, Gustafson AM, Zhang X (2009). MicroRNAs as modulators of smoking-induced gene expression changes I human airway epithelium.. Proc Natl Acad Sci U S A.

[41] Izzotti A, Larghero P, Cartiglia C, Longobardi M, Pfeffer U (2010). Modulation of microRNA expression by budesonide, phenethyl isothiocyanate and cigarette smoke in mouse liver and lung.. Carcinogenesis.

[42] Guerrero-Preston R, Goldman LR, Brebi-Mieville P, Ili-Ganga C, Lebron C (2010). Global hypomethylation is associated with in utero exposure to cotinine and perfluorinated alkyl compounds.. Epigenetics.

[43] Breton CV, Byun HM, Wenten M, Pan F, Yang A (2009). Prenatal tobacco smoke exposure affects global and gene-specific DNA methylation.. Am J Respir Crit Care Med.

[44] Sauer J, Jang H, Zimmerly EM, Kim KC (2010). Agening, chronic alcohol consumption and folate are determinnats of genomic DNAmethylation, p16 promoter methylation and the expression of p16 in the mouse colon.. Br J Nutr.

[45] Seitz HK, Stickel F (2007). Molecular mechanisms of alcohol-mediated carcinogenesis.. Nat Rev Cancer.

[46] Pascual M, Boix J, Felipo V, Guerri C (2009). Repeated alcohol administration during adolescence causes changes in the mesolimbic dopaminergic and glutamatergic systems and promotes alcohol intake in the adult rat.. J Neurochem.

[47] Miranda RC, Pietrzykowski AZ, Tang Y, Sathyan P, Mayfield D (2010). MicroRNAs: master regulators of ethanol abuse and toxicity?. Alcohol Clin Exp Res.

[48] Tarantini L, Bonzini M, Apostoli P, Pegoraro V, Bollati V (2009). Effects of particulate matter on genomic DNA methylation content and iNOS promter methylation.. Environ Health Perspect.

[49] Bollati V, Baccarelli A, Hou L, Bonzini M, Fustinoni S (2007). Changes in DNA methylation patterns in subjects exposed to low-dose benzene.. Cancer Res.

[50] Baccarelli A, Wright RO, Bollati V, Tarantini L, Litonjua AA (2009). Rapid DNA methylation changes after exposure to traffic particles.. Am J Respir Crit Care Med.

[51] Wilker EH, Baccarelli A, Suh H, Vokonas P, Wright RO (2010). Black carbon exposures, blood pressure and interactions with single nucleotide polymorphisms in microRNA processing genes.. Environ Health Perspect.

[52] Shin CM, Kim N, Jung Y, Park JH, Kang GH (2010). The role of Helicobacter pylori infection in aberrant DNA methylation along multistep gastric carcinogenesis.. Cancer Sci.

[53] Tsai CN, Tsai CL, Tse KP, Chang HY, Chang YS (2002). The Epstein-Barr virus oncogene product, latent membrane protein 1, induces the downregulation of E-cadherin gene expression via activation of DNA methyltransferases.. Proc Natl Acad Sci U S A.

[54] Backdahl L, Bushell A, Beck S (2009). Inflammatory signalling as mediator of epigenetic modulation in tissue-specific chronic inflammation.. Int J Biochem Cell Biol.

[55] Medzhitov R, Horng T (2009). Transcriptional control of the inflammatory response.. Nature Rev Immunol.

[56] Weaver IC, Cervoni N, Champagne FA, D'Alessio AC, Sharma S (2004). Epigenetic programming by maternal behaviour.. Nat Neurosci.

[57] McGee SL, Fairlee E, Graham AP, Hargreaves M (2009). Exercise-induced histone modifications in human skeletal muscle.. J Physiol.

[58] Radom Aizik S, Zaldivar FP, Oliver SR, Galassetti PR, Cooper DM (2010). Evidence for microRNA involvement in exercise-associated neutrophil gene expression changes.. J Appl Physiol.

[59] Kadota M, Yang HH, Hu N, Wang C, Hu Y (2007). Allele-specific chromatin immunoprecipitation studies show genetic influence on chromatin state in human genome.. PLoS Genet.

[60] Wong CC, Caspi A, Williams B, Craig IW, Houts R (2010). A longitudinal study of epigenetic variation in twins.. Epigenetics.

[61] Bjornsson HT, Sigurdsson MI, Fallin MD, Irizarry RA, Aspelund T (2008). Intra-individual change over time in DNA methylation with familial clustering.. JAMA.

[62] French HJ, Attenborough R, Hardy K, Shannon F, Williams RBH (2009). Interindividual variation in epigenomic phenomena in humans.. Mamm Genome.

[63] Heijmans BT, Kremer D, Tobi EW, Boomsa DI, Slagboom PE (2007). Heritable rather than age-related and stochastic factors dominate variation in DNA methylation of the human IGF2/H19 locus.. Hum Mol Genet.

[64] Zhang D, Cheng L, Badner JA, Chen C, Chen Q (2010). Genetic control of individual differences in gene-specific methylation in human brain.. Am J Hum Genet.

[65] Dimas AS, Dermitzakis ET (2009). Genetic variation of regulatory systems.. Curr Opin Genet Dev.

[66] Kong A, Steinthorsdottir V, Masson G, Thorleifsson G, Sulem P (2009). Parental origin of sequence variants associated with complex diseases.. Nature.

[67] Davey Smith G, Lawlor DA, Harbord R, Timpson N, Day I (2007). Clustered environments and randomized genes: a fundamental distinction between conventional and genetic epidemiology.. PLoS Med.

[68] Davey Smith G, Ebrahim S (2003). ‘Mendelian randomization’: can genetic epidemiology contribute to understanding environmental determinants of disease?. Int J Epidemiol.

[69] Davey Smith G (2010). Mendelian randomization for strengthening causal inference in observational studies: applications to gene by environment interaction.. Perspect Psychol Sci.

[70] Sheehan NA, Didelez V, Burton PR, Tobin MD (2008). Mendelian randomization and causal inference in observational epidemiology.. PLoS Med.

[71] Lewis SJ, Davey Smith G (2005). Alcohol, ALDH2 and esophageal cancer: a meta-analysis which illustrates the potentials and limitations of a Mendelian randomization approach.. Cancer Epidemiol Biomarkers Prev.

[72] Boccia S, Hashibe M, Galli P, De Feo E, Asakage T (2009). Aldehyde dehydrogenase 2 and head and neck cancer: a meta-analysis implementing a Mendelian randomization approach.. Cancer Epidemiol Biomarkers Prev.

[73] Chen L, Davey Smith G, Harbord R, Lewis S (2008). Alcohol intake and blood pressure: a systematic review implementing Mendelian randomization approach.. PLoS Med.

[74] Linsel-Nitschke P, Gotz A, Erdmann J, Braenne I, Braund P (2008). Lifelong reduction of LDL-cholesterol related to a common variant in the LDL-receptor gene decreases the risk of coronary artery disease–a Mendelian randomization study.. PLoS ONE.

[75] Teslovich M, Musunumu K, Smith AV, Edmondson AC, Stylianou IM (2010). Biological, clinical and population relevance of 95 loci for blood lipids.. Nature.

[76] Schuldiner AR, Pollin TI (2010). Variation in blood lipids.. Nature.

[77] Freathy RM, Timpson NJ, Lawlor DA, Pouta A, Ben-Shlomo Y (2008). Common variation in the FTO gene alters diabetes-related metabolic traits to extent expected, given its effect on BMI.. Diabetes.

[78] Timpson N, Harbord R, Davey Smith G, Zacho J, Tybaerg-Hansen A (2009). Does greater adiposity increase blood pressure and hypertension risk? Mendelian randomization using Fto/Mc4r genotype.. Hypertension.

[79] Timpson NJ, Sayers A, Davey Smith G, Tobias JH (2002). How does body fat influence bone mass in childhood? A Mendelian randomisation approach.. J Bone Miner Res.

[80] Timpson NJ, Lawlor DA, Harbord RM, Gaunt TR, Day INM (2005). C-reactive protein and its role in metabolic syndrome: Mendelian randomisation study.. Lancet.

[81] Zacho J, Tybjoerg-Hansen A, Jensen JS, Grande P, Sillensen H (2008). Genetically elevated C-reactive protein and ischaemic vascular disease.. New Engl J Med.

[82] Emilsson V, Thorleifsson G, Zhang B, Leonardson AS, Zink F (2008). Genetics of gene expression and its effect on disease.. Nature.

[83] Li H, Lu L, Manly KF, Chesler EJ, Bao L (2005). Inferring gene transcriptional modulatory relations: a genetical genomics approach.. Hum Mol Genet.

[84] Gilbert SF, Epel D (2009). Ecological developmental biology: Integrating epigenetics, medicine and evolution.

[85] Liu Z, Sall A, Yang D (2008). MicroRNA: an emerging therapeutic target and intervention tool.. Int J Mol Sci.

[86] Abbott A (2010). Project set to map marks on genome.. Nature.

